# Epidemiology and Clinical Features of Listeriosis in Gipuzkoa, Spain, 2010–2020

**DOI:** 10.3389/fmicb.2022.894334

**Published:** 2022-06-09

**Authors:** Pedro Vallejo, Gustavo Cilla, Maddi López-Olaizola, Diego Vicente, José María Marimón

**Affiliations:** ^1^Microbiology Department, Osakidetza Basque Health Service, Donostialdea Integrated Health Organization, San Sebastián, Spain; ^2^Department of Preventive Medicine, University of the Basque Country (UPV/EHU), San Sebastián, Spain; ^3^Microbiology Department, Infectious Diseases Area, Biodonostia Health Research Institute, Vaccine Preventable Diseases Group, Osakidetza Basque Health Service, Donostialdea Integrated Health Organization, San Sebastián, Spain; ^4^Microbiology Department, Infectious Diseases Area, Respiratory Infection and Antimicrobial Resistance Group, Biodonostia Health Research Institute, Osakidetza Basque Health Service, Donostialdea Integrated Health Organization, San Sebastián, Spain

**Keywords:** *Listeria monocytogenes*, listeriosis, zoonosis, food - borne pathogens, MLST (multilocus sequence typing)

## Abstract

**Background:**

Listeriosis continues to be one of the most important notifiable foodborne diseases. Nonetheless, in Spain, there are few data on the molecular epidemiology of *Listeria monocytogenes* infections in recent years.

**Aim:**

To describe clinical features and the molecular epidemiology of human listeriosis over an 11-year period (2010–2020) in Gipuzkoa, Northern Spain.

**Methods:**

A total of 111 isolates, all but one from invasive disease, were studied. Serotyping (agglutination and multiplex polymerase chain reaction [PCR]) and multilocus sequence typing were performed for all isolates. Antibiotic susceptibility was assessed by the broth microdilution method.

**Results:**

The average annual incidence of listeriosis in non-pregnancy-associated cases was 1.55 per 100,000 population, with a 1-month mortality rate of 22.2%. In pregnant women, the average incidence was 0.45 cases per 1,000 pregnancies. Twenty-four sequence types were identified, serotype 4b ST1 (24.3%) being the most frequent followed by 1/2b ST87 (18.9%), which caused two long outbreaks in 2013–2014. A significant association was observed between ST219 and meningitis (*p* < 0.001). All isolates were susceptible to ampicillin as well as other antibiotics used in listeriosis treatment.

**Conclusion:**

Despite current control measures, listeriosis continues to be an important cause of mortality in the elderly, preterm birth, and miscarriages in pregnant women. Improvements in the control and diagnosis of listeriosis are needed to reduce the impact of this infection on vulnerable populations.

## Introduction

Listeriosis, a zoonotic disease caused by *Listeria monocytogenes*, is a major public health problem and one of the most common notifiable foodborne diseases. Most diagnosed cases of listeriosis correspond to sepsis, meningitis, and perinatal infections, but a small portion of cases are other invasive forms like endophthalmitis, endocarditis, and periprosthetic joint infection (Gori et al., 2020). *L. monocytogenes* also causes non-invasive gastrointestinal disease that is probably underdiagnosed as it is not included in routine stool cultures or as a target in the main multiplex molecular panels used for the diagnosis of gastrointestinal infections (Binnicker, 2015).

The great majority of cases of human listeriosis are sporadic but outbreaks of listeriosis due to contaminated food are not unusual. In Spain, the prevention of listeriosis is regulated by the European microbiological food safety criteria, which indicate that *L. monocytogenes* must not be presented in 25 g of sample in the case of products intended for infants or special medical purposes but permit a maximum limit of 100 colony forming units per gram during the shelf life of other products (EUR-Lex, 2020). Despite these control measures, *L. monocytogenes* is still one of the most important foodborne pathogens in Europe and the most severe zoonotic diseases, with the highest hospitalization and case-fatality rates (EFSA, 2019). In Spain, between 1997 and 2015, hospitalizations due to listeriosis showed an increasing trend and were more frequent in the north of the country (Herrador et al., 2019). Listeriosis in Spain has been a notifiable disease since 2015 (Ministerio de Sanidad, 2015).

Listeriosis outcome is greatly influenced by host immune status, patients with underlying conditions such as cancer, diabetes, AIDS, and other immunocompromising illnesses being more likely to be hospitalized (Goulet et al., 2012). In the general population, people older than 65 years are the most susceptible, with a case-fatality rate of around 17% in the European Union (EU) (EFSA, 2019). The other important groups of susceptible patients are pregnant women. The presentation of listeriosis during pregnancy includes flu-like symptoms such as fever, headache, and gastrointestinal discomfort, but notably, listeriosis is asymptomatic in a third of infected pregnant women. On the other hand, clinical features of neonatal listeriosis are similar to group B streptococcal infection: respiratory distress, fever, cyanosis, hypotension, and lethargy (Lamont et al., 2011). In a large prospective French study (the MONALISA cohort), almost all pregnant women with listeriosis experienced fetal or neonatal complications (Charlier et al., 2017).

Traditionally, *L. monocytogenes* has been detected by microbiological culture. More recently, polymerase chain reaction (PCR)-based methods have become available for the detection of this pathogen alone or in multiplex PCR. Epidemiology has been commonly based on serotyping, with four serotypes, 1/2a, 1/2b, 1/2c, and 4b recognized in most human infections (ECDC et al., 2021). Molecular epidemiology research involving the subtyping of isolates is critical for studying the behavior of *L. monocytogenes* in the environment. Commonly used subtyping methods are pulsed-field gel electrophoresis (PFGE) and multilocus sequence typing (MLST) (Jadhav et al., 2012). Currently, whole-genome sequencing is the technique of choice for epidemiologic studies (Jackson et al., 2016; Lüth et al., 2018; Van Walle et al., 2018).

The main objective of this study was to analyze the epidemiology and clinical outcome of invasive listeriosis in Gipuzkoa, northern Spain, between 2010 and 2020 and to determine the microbiological characteristics of *L. monocytogenes* invasive isolates.

## Materials and Methods

This retrospective study was conducted in Donostia University Hospital (DUH), located in Donostia-San Sebastián, the capital city of the province of Gipuzkoa, Basque Country, in the north of Spain. During the study period, the hospital catchment population was between 417,347 and 427,416 people according to the official census data of 2010 and 2020, respectively, representing nearly 60% of the overall population of Gipuzkoa (720,458 people in 2020) (EUSTAT, 2021).

For establishing incidence data and clinical characteristics of the infection, all cases of listeriosis diagnosed by culture in the Microbiology Department at DUH from 2010 to 2020 were included in the study. In the same years, 18 cases of *L. monocytogenes* isolated at the three county hospitals in the province of Gipuzkoa were also sent to the DUH for molecular characterization. As these isolates were not received on a systematic basis, they were only included in the study of microbiological characteristics of *L. monocytogenes* isolates. A pregnancy-associated episode was considered only one case, regardless of whether the isolates were collected from the mother, the child, or both.

### Microbiological Procedures

*Listeria monocytogenes* were isolated from blood cultures using the BACTEC FX blood culture system (BD, New Jersey, United States). Samples from other common sterile localizations were cultured on Columbia Agar with 5% Sheep Blood (agar COS, BD) and incubated at 35°C. Investigation of *L. monocytogenes* in feces was performed using Listeria selective-chromogenic agar (as per Ottaviani and Agosti) (RPD, Barcelona, Spain).

### Identification

Before 2013, colonies with a typical morphological appearance of *L. monocytogenes* (small, smooth, translucent grayish colonies with a narrow beta-hemolysis in blood-agar) were Gram-stained, and Gram-positive bacilli or coccobacilli were then identified using the API20 Corynebacterium System (bioMérieux, France). Since 2013, all isolates have been identified by matrix-assisted laser desorption/ionization time-of-flight mass spectrometer analysis (Bruker Daltonik, Germany). A score of >2.0 was used as the threshold for species identification.

### Antibiotic Susceptibility

Antibiotic susceptibility was assessed by the broth dilution method according to the European Committee on Antimicrobial Susceptibility Testing (EUCAST) guidelines and clinical breakpoints (EUCAST, 2022). The antibiotics tested were penicillin, ampicillin, amoxicillin-clavulanic acid, meropenem, erythromycin, trimethoprim-sulfamethoxazole, and levofloxacin.

### Typing

Serotyping was performed by slide agglutination using Listeria-O-Antisera (Difco; BD Diagnostics, Sparks, MD, United States) and multiplex PCR as previously described (Doumith et al., 2004). *L. monocytogenes* isolates were genotyped according to the MLST scheme using the primers and conditions described by the Pasteur Institute (Moura et al., 2016).

### Statistical Analysis

Categorical data were compared with the Fisher’s exact test using Prism 7.05, GraphPad software^1^. A *p*-value < 0.05 was considered statistically significant.

### Ethical Considerations

As the study was retrospective, informed consent was not required from patients. Patient data were handled in accordance with Spanish data protection laws and regulations in force (Organic Law 3/2018, of December 5, on the Protection of Personal Data and guarantee of digital rights).

## Results

Between 2010 and 2020, 93 cases of listeriosis were diagnosed at the DUH, all in hospitalized patients (Supplementary Table 1), and of these, 21 (22.6%) were pregnancy associated. The average annual incidence was 2.0 cases per 100,000 population (range: 1.17 in 2019 to 3.32 in 2013) (Table 1).

**TABLE 1:** Annual distribution (number of cases) and incidence per 100,000 population of non-pregnancy-associated cases of human listeriosis in Gipuzkoa, north of Spain 2010–2020.

Age groups		2010	2011	2012	2013	2014	2015	2016	2017	2018	2019	2020	Total cases	Average annual incidence
20-64 years	W	2	0	1	2	1	2	0	2	0	0	0	10	0.72
	M	2	2	1	2	1	1	0	1	0	1	1	12	0.86
65-79 years	W	2	0	0	2	0	0	0	1	1	0	1	7	1.74
	M	0	2	1	2	3	2	3	0	4	0	2	19	6.03
≥ 80 years	W	1	2	0	1	0	0	2	0	0	1	1	8	3.79
	M	0	1	1	1	2	1	4	0	5	1	0	16	14.21
Total^1^		7	7	4	10	7	6	9	4	10	3	5	72	1.55
Total^2^		8	10	8	14	10	7	9	5	11	5	6	93	2.00

*W: women; M: men. 1. Total, excluding pregnancy associated cases. 2. Total, including pregnancy associated cases.*

### Non-Pregnancy-Associated Listeriosis

There were 72 (77.4%) non-pregnancy-associated cases of listeriosis in patients with a mean age of 71.3 years (range: 33–91 years), and it was more frequent in men (47 cases, 65.3%). In the 20–64 years group, there was no marked difference between sexes (incidence of 0.72 in men and 0.86 in women) but in >65-years old, the average annual incidence was 3.4-fold higher in men (8.18 in men *vs*. 2.42 in women). Men older than 80 years showed the highest incidence rate, with 14.2 cases per 100,000 people (16 cases, 22.2%).

*Listeria monocytogenes* caused sepsis in 49 (68%) patients and meningitis in 17 (23.6%) patients (8 of them also with sepsis), the other cases being 2 of peritonitis, 1 of spondylodiscitis, 1 of endophthalmitis, 1 of urinary infection, and 1 of acute gastroenteritis.

Most patients (61, 84.7%) had relevant underlying conditions: 37 (60.7%) were immunosuppressed (chemotherapy or immunosuppressive treatments) and 20 (32.8%) had diabetes mellitus, high blood pressure, or hyperlipidemia.

Overall, 1-month and 1-year mortality rates were 22.2% (16 patients) and 41.7% (30 patients), respectively. Patients who died in the first month after infection had a mean age of 73.4 years (range: 54–91 years). Nonetheless, no statistical associations were found between age older than 65 and 1-month or 1-year mortality (*p* = 0.76 and *p* = 0.61, respectively). Further, no differences were observed between women and men in 1-month or 1-year mortality, either overall or in age groups (20–64 years, 65–79 years and > 80 years, *p* > 0.05 in all cases).

Of the 30 patients who died in the first year after infection, 21 were immunosuppressed (20 being under treatment for cancer and 1 having monoclonal gammopathy), 4 had diabetes mellitus and/or high blood pressure, 1 has myasthenia gravis, and 1 had chronic renal failure. A 1-month mortality was not associated with immunosuppression (*p* = 0.41), but 1-year mortality was associated with immunosuppressed status (*p* = 0.009). No association was found between meningitis and 1-month or 1-year mortality, age older than 65 years or immunosuppression (*p* > 0.05 for all of them).

### Pregnancy-Associated Listeriosis

Twenty-one pregnancy-associated cases affected 46 patients (21 mothers and 25 fetuses). Cases were diagnosed in 13 blood cultures (10 from mothers and 3 from neonates), 7 placental cultures, and 1 otic exudate of a neonate. The estimated incidence of pregnancy-associated listeriosis throughout the study period was 0.45 cases per 1,000 pregnancies (range from 0 in 2016 to 0.93 in 2013).

None of the 21 pregnant women had any relevant underlying diseases, and one was admitted to the intensive care unit for severe sepsis. Five (20%) fetal losses occurred: two abortions and three stillbirths. Nine (42.9%) pregnancies, including two twin pregnancies, ended in preterm births (range 28–35 weeks of pregnancy). Two of the premature neonates suffered sequelae: obstructive hydrocephalus due to meningitis and dolichocephaly with the absence of temporoparietal sutures. None of the pregnant women died due to listeriosis.

### Serotypes and Genotypes

Of the total of 111 episodes of listeriosis (93 from DUH and 18 from the three-county hospitals), serotype 4b was the most common (53.2%, 59 isolates) followed by 1/2b (27%, 30 isolates), 1/2a (18.9%, 21 isolates), and 1/2c (1 isolate) (Table 2). Excluding pregnancy-associated cases, infection with serotype 4b was associated with 1-year mortality (*p* = 0.042). There were no associations of any serotypes with meningitis or perinatal infection (*p* > 0.05 in all cases).

**TABLE 2:** Distribution of lineages, serotypes, clonal complexes (CCs), and sequence types (STs) of *L. monocytogenes* isolates in Gipuzkoa, 2010–2020.

Lineage	Serotype	CC	ST	Total isolates
I	1/2b	3	3	8
I	1/2b	87	87	21
I	4b	1	1	27
I	4b	1	2892^a^	1
I	4b	4	4	4
I	4b	6	6	7
I	4b	213	213	4
I	4b	4	219	9
II	1/2a	8	8	7
II	1/2a	101	431	4
Others	-	-	-	19
Total	-	-	-	111

*^a^ST892 is a new ST, a single locus variant of ST1.*

**FIGURE 1:**
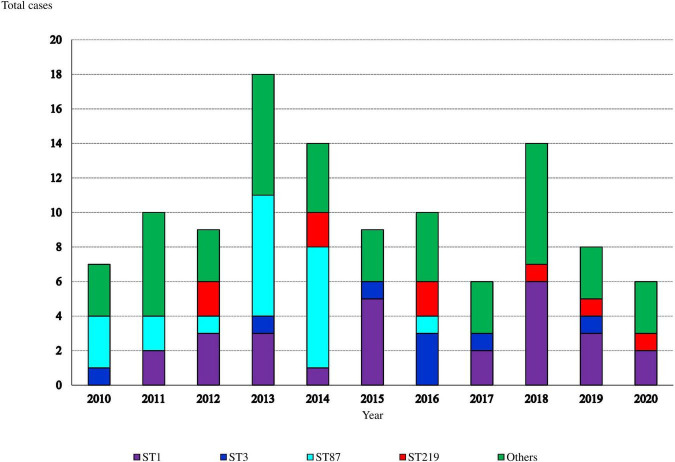
Annual distribution of sequence-types (STs) causing invasive listeriosis (such as pregnancy and non-pregnancy associated cases).

Ninety (81%) isolates belonged to lineage I and 21 (19%) to lineage II. Each sequence type (ST) always belonged to the same serotype (Supplementary Table 2). Serotype 4b/ST1 was the most frequent (24.3%, 27 isolates), followed by 1/2b/ST87 (18.9%, 21 isolates) and 4b/ST219 (8.1%, 9 isolates). The distribution of ST1 isolates throughout the study period was homogeneous (Figure 1). In contrast, 95% of ST87 isolates were more common between 2010 and 2014, particularly in 2013 and 2014 associated with two outbreaks of two different strains of the same ST, ST87 (Pérez-Trallero et al., 2014).

Three of the four cases of ST431 (CC101) were isolated in 2016 (Supplementary Table 3), but they could not be epidemiologically linked. The new ST2892, a single locus variant of ST1, was isolated in 2014.

Most cases of perinatal infections were caused by ST1 (41.7%, 10 isolates) and ST87 (37.5%, 9 isolates), both STs being associated with pregnancy (*p* = 0.029 and *p* = 0.015).

The ST1 isolates caused one abortion, two stillbirths, and five preterm births, while ST87 isolates were responsible for one abortion and three preterm births. No statistically significant associations were observed between any STs and fetal disease.

Further, in non-perinatal listeriosis, ST1 and ST87 were the most frequent (19.5 and 13.8% of cases, respectively). Ten patients infected with ST1 *L. monocytogenes* died, three of them within the first month after diagnosis. In ST87 infected cases, six patients died, two in the first month. No significant associations were found between ST1 or ST87 and death.

In the non-pregnancy associated group, there was a strong association between meningitis and ST219 (*p* < 0.001), this ST causing 7 of 17 cases (41.2%). In contrast, none of the four infections with ST4 (that also belongs to CC4) caused meningitis.

### Antibiotic Susceptibility

All *L. monocytogenes* isolates were susceptible to all antibiotics tested (Table 3).

**TABLE 3:** Minimum inhibitory concentration (MIC) range, MIC_50_ and MIC_90_ of 111 *L. monocytogenes* isolates.

Antibiotic	MIC range (mg/L)	MIC50 (mg/L)	MIC90 (mg/L)	%S
Penicillin	0.03-1	0.25	0.50	100
Ampicillin	≤ 0.12-1	0.50	0.50	100
Amoxicillin - clavulanic acid	≤ 0.50/0.25	≤0.50/0.25	≤ 0.50/0.25	100
Meropenem	≤ 0.25	≤0.25	≤ 0.25	100
Erythromycin	≤ 0.25- > 16	≤0.25	≤ 0.25	100
Trimethoprim-sulfamethoxazole	≤ 0.5/9.5	≤0.5/9.5	≤ 0.5/9.5	100^a^
Levofloxacin	0.5-4	2	2	NA

*NA = EUCAST breakpoints not available; ^a^For trimethoprim-sulfamethoxazole, Clinical & Laboratory Standards Institute (CLSI) clinical breakpoints were used.*

## Discussion

Listeriosis is a notifiable disease and EU health authorities have implemented control measures in the food chain to contain the transmission of this pathogen to humans (Reglamento (CE) No 2073/2005, 2005 of 15 November 2005 on microbiological criteria for foodstuffs [Text with EEA relevance], EUR-Lex, 2020). Despite these efforts, the rates of human listeriosis between 2014 and 2018 in the EU have remained between 0.43 and 0.48 cases per 100,000 population. In Spain, in 2018, the incidence of listeriosis reported was 0.89 cases per 100,000 people (EFSA, 2019). Notably, the overall incidence of listeriosis in Gipuzkoa between 2010 and 2020 (2 cases per 100,000 people) was higher than in places with the highest previously reported incidence rates in Europe, Spain overall, and even other Spanish regions (Zamora-López et al., 2015; Muñoz-Gallego et al., 2017; Herrador et al., 2019; Beamonte Vela et al., 2020). This high incidence compared to other regions could be influenced, among other factors, by the eating habits of the population in our province. In particular, several studies have concluded that some outbreaks in the Basque Country have been related to the consumption of foie gras (Pérez-Trallero et al., 2014) or fresh cheese (de Castro et al., 2012) and have also identified the presence of *L. monocytogenes* in ready-to-eat foods, such as smoked fish (Garrido et al., 2009; Chau et al., 2017).

We also observed a pregnancy-associated listeriosis rate of 0.45 cases per 1,000 pregnancies, higher than in other studies (Filipello et al., 2017; Craig et al., 2019). The rate of perinatal listeriosis is likely to be higher than reported as it has been seen that up to 45% of maternal blood cultures are negative in cases of perinatal listeriosis (Charlier et al., 2017). Only a third of the listeriosis cases in pregnancy did not have severe consequences for the mother or fetus. Our study revealed perinatal death in 20% and preterm birth in 42.9% of cases. These data are consistent with large studies of maternal listeriosis, in which approximately 20–24% of cases resulted in abortion or stillbirth, and 45% in preterm births (Mylonakis et al., 2002; Charlier et al., 2017).

Overall, listeriosis mainly affected elderly people, especially men, and was associated with a high mortality rate. In non-pregnancy-associated cases, the median age was 71.3 years, similar to that found in Denmark between 2002 and 2012 (Jensen et al., 2016). The 1-month mortality rate was 22.2%, higher than the 13% found in a German study of 5,576 cases between 2010 and 2019 (Wilking et al., 2021), but the same as in Denmark (Jensen et al., 2016) and lower than the 39.7% in another Spanish study (Muñoz-Gallego et al., 2017). In the MONALISA cohort, the 3-month mortality rate in bacteremic listeriosis was 46% (Charlier et al., 2017), similar to that for 1-year mortality in our population (41.7%). A significant association was found between immunosuppression and 1-year mortality, indicating that invasive listeriosis mainly affects patients with a short-life expectancy. In Europe, a 31% increase in the number of listeriosis deaths was observed between 2018 and 2019, partially explained by the aging population and increases in the numbers of immunosuppressed patients (EFSA, 2021).

Despite the outbreaks caused by serotype 1/2b in 2013–2014, serotype 4b was the most frequent in our region, unlike in the European Listeria typing exercise (ELiTE) report (ECDC et al., 2021), in which the most common serotype in human infections between 2010 and 2011 was 1/2a. The distribution of STs can vary considerably in different countries. Excluding ST87 (CC87) isolates, the most abundant STs in our region were ST1 (CC1), ST219 (CC4), ST3 (CC3), and ST8 (CC8). In contrast, ST8, ST2, and ST6 were the most abundant in the Danish study of 387 isolates (Jensen et al., 2016), and isolates belonging to CC1, CC2, CC4, and CC6 dominated in France (Charlier et al., 2017). In the south of Spain (Andalucía), a large outbreak of Listeriosis 4b/ST388 involving 219 people occurred between July and September 2019, associated with the consumption of shredded meat (“*carne mechada*”) (Ministerio de Sanidad, 2019). Despite the wide extent of this outbreak, this ST was not detected in 2019 in Gipuzkoa, again highlighting the importance of the eating habits of each region in listeriosis.

An interesting phenomenon occurred with ST87. This ST, considered hypervirulent (as it carries *L. monocytogenes* pathogenicity island-4 (Maury et al., 2016), has been documented many times in studies in Taiwan (Huang et al., 2021) and China (Zhang et al., 2019; Chen et al., 2020), being one of the most common STs in food and human infections in these countries. Besides, most ST87 isolates in the MLST database (accessed on February 28, 2022) are from Asia. In Spain, a case of sepsis due to *L. monocytogenes* ST87 was diagnosed in 2012 in León, northern Spain (Ariza-Miguel et al., 2015), and in 2013–2014, there were two outbreaks in our region (Pérez-Trallero et al., 2014). A study of food confiscated from passengers arriving in 2012 and 2013 at Bilbao airport in the neighboring province of Vizcaya, Spain (Rodríguez-Lázaro et al., 2015) revealed that some products were contaminated with *L. monocytogenes*. Specifically, two products coming from South America (Colombia and Venezuela) carried *L. monocytogenes* ST87. Moreover, other Spanish investigations have detected *L. monocytogenes* ST87 in poultry processing facilities in 2012–2013 (Melero et al., 2019) and food processing premises (Manso et al., 2019) indicating that ST87 was well established in our environment. Excluding China, *L. monocytogenes* ST87 is rarely reported as a cause of human infection, however, it only having appeared in the Czech Republic (Hluchanova et al., 2022), France (Moura et al., 2017), and South Africa (Smith et al., 2019). Nonetheless, some studies have detected the presence of ST87 in food industry facilities in Austria in 2014 (Rückerl et al., 2014) and Poland in 2019 (Sosnowski et al., 2019). Other research also detected *L. monocytogenes* ST87 in Chinese food confiscated at an Austrian airport (Schoder et al., 2015).

Another notable finding concerns ST219. To our knowledge, only one clinical case has been described in the literature, a case of meningoencephalitis in Italy in 2018 (Sotgiu et al., 2018). The other publications in which ST219 appears report studies of the presence of *L. monocytogenes* in cattle and food processing plants (Jorgensen et al., 2021; Palacios-Gorba et al., 2021). We describe here a series of human ST219 cases, well distributed across the 11 years of the study. We found a strong association between ST219 (serotype 4b, lineage I) and meningitis. It has been seen that ST219 belongs to the hypervirulent clone CC4 (Manso et al., 2019), like ST4, also considered hypervirulent as it carries *L. monocytogenes* pathogenicity island-4 (Raschle et al., 2021). The CC4 was also associated with neurolisteriosis and pregnancy-related cases in the MONALISA cohort (Charlier et al., 2017). In our study, all CC4 meningitis corresponded to ST219 isolates and there were no cases of meningitis caused by ST4. We are currently conducting molecular studies based on whole-genome sequencing to elucidate the genetic determinants of ST219 tropism for the CNS.

In Gipuzkoa, ST1 (CC1) and ST87 (CC87) were associated with perinatal infections. ST1 has been considered a hypervirulent ST, as it carries putative virulence factors (Manso et al., 2019). Other studies have also related ST1 and ST87 to perinatal infections (Zhang et al., 2019), ST87 being the most common genotype of perinatal listeriosis in Taiwan (Huang et al., 2021).

No antibiotic resistance against commonly used first-line treatments of listeriosis was observed, as in other studies (Ariza-Miguel et al., 2015; Madeo et al., 2015). At present, antibiotic resistance is not a matter of concern in *L. monocytogenes* although monitoring is important due to the high severity of many infections by this pathogen.

The incidence of listeriosis in Europe based on case notifications is very low compared to that of other infectious diseases, although the real incidence is, according to our study, underestimated. Nevertheless, the burden of the disease, especially mortality in the elderly and miscarriages in pregnant women makes continued surveillance of *L. monocytogenes* infections necessary. More control measures or more restrictive limits on contaminated foods are needed to prevent this zoonotic disease in susceptible populations. Studies on the molecular epidemiology of listeriosis will improve our understanding of the spreading and characteristics of the disease caused by virulent clones, with the goal of reducing the incidence and clinical impact of the infection.

## Data Availability Statement

The original contributions presented in the study are included in the article/Supplementary Material, further inquiries can be directed to the corresponding author.

## Ethics Statement

Ethical review and approval was not required for the study on human participants in accordance with the local legislation and institutional requirements. Written informed consent from the participants’ legal guardian/next of kin was not required to participate in this study in accordance with the national legislation and the institutional requirements.

## Author Contributions

PV was responsible for the management of the epidemiological surveillance data. PV and JM were in charge of the conception and design of the study and wrote the first draft of the manuscript. GC, ML-O, and DV contributed to the study data analysis and interpretation also providing technical support. All authors contributed to the review of the different drafts and approved all versions of the manuscript.

## Conflict of Interest

The authors declare that the research was conducted in the absence of any commercial or financial relationships that could be construed as a potential conflict of interest.

## Publisher’s Note

All claims expressed in this article are solely those of the authors and do not necessarily represent those of their affiliated organizations, or those of the publisher, the editors and the reviewers. Any product that may be evaluated in this article, or claim that may be made by its manufacturer, is not guaranteed or endorsed by the publisher.

## Footnotes

1
https://www.graphpad.com/scientific-software/prism/
