# Supplementary material for: Epidemiology and Clinical Features of Listeriosis in Gipuzkoa, Spain, 2010–2020
Source: Front Microbiol. 2022 Jun 9;13:894334. doi: 10.3389/fmicb.2022.894334 (PMC9218358; doi:10.3389/fmicb.2022.894334)
Supplement: Supplementary file 1 [file Table_1.docx]

Table S1. Cases and epidemiological, clinical, and molecular data

| Case | Year | Age | Gender | Source | Serotype | ST^a^ | CC^b^ | Death^c^ | Time to death^d^ | Immunosuppression | Pregnancy-associated | Meningitis |
| --- | --- | --- | --- | --- | --- | --- | --- | --- | --- | --- | --- | --- |
| 1 | 2010 | 75 | Female | Peritoneal fluid | NA | NA | NA | No |  | No | No | No |
| 2 | 2010 | 58 | Female | Blood | 1b | 87 | 87 | Yes | One year | No | No | No |
| 3 | 2010 | 32 | Female | Blood | 1b | 87 | 87 | No |  | No | Yes | No |
| 4 | 2010 | 62 | Male | Blood | 4b | 2 | 2 | Yes | One month | No | No | No |
| 5 | 2010 | 57 | Female | Blood | 4b | 6 | 6 | Yes | One year | No | No | No |
| 6 | 2010 | 63 | Male | Blood | 1b | 3 | 3 | No |  | Yes | No | No |
| 7 | 2010 | 87 | Female | Blood | 1b | 87 | 87 | Yes | One month | No | No | No |
| 8 | 2010 | 66 | Female | CSF | 1a | 8 | 8 | No |  | No | No | Yes |
| 9 | 2011 | 77 | Female | Blood | 4b | 217 | 217 | Yes | One month | Yes | No | No |
| 10 | 2011 | 27 | Female | Blood | 4b | 1 | 1 | No |  | No | Yes | No |
| 11 | 2011 | 54 | Male | Blood | 4b | 1 | 1 | Yes | One month | Yes | No | No |
| 12 | 2011 | 72 | Male | Blood | 1a | 18 | 18 | Yes | One year | Yes | No | No |
| 13 | 2011 | 63 | Male | Blood | 1b | 87 | 87 | No |  | Yes | No | No |
| 14 | 2011 | 71 | Male | Blood | 4b | 213 | 213 | No |  | Yes | No | No |
| 15 | 2011 | 85 | Female | CSF | 4b | 213 | 213 | No |  | No | No | Yes |
| 16 | 2011 | 0 | Male | Blood | 1a | 14 | 14 | No |  | No | Yes | No |
| 17 | 2011 | 37 | Female | Blood | 1b | 87 | 87 | No |  | No | Yes | No |
| 18 | 2011 | 89 | Male | Blood | 4b | 4 | 4 | Yes | One year | Yes | No | No |
| 19 | 2012 | 61 | Female | Blood | 4b | 1 | 1 | Yes | One year | Yes | No | No |
| 20 | 2012 | 73 | Male | Blood | 4b | 54 | 54 | Yes | One month | Yes | No | No |
| 21 | 2012 | 85 | Male | Blood | 4b | 6 | 6 | No |  | No | No | No |
| 22 | 2012 | 61 | Male | CSF | 4b | 219 | 4 | No |  | No | No | Yes |
| 23 | 2012 | 32 | Female | Placenta | 4b | 219 | 4 | No |  | No | Yes | No |
| 24 | 2012 | 32 | Female | Blood | 1/2c | 9 | 9 | No |  | No | Yes | No |
| 25 | 2012 | 41 | Female | Placenta | 4b | 1 | 1 | No |  | No | Yes | No |
| 26 | 2012 | 0 | Male | Blood | 4b | 1 | 1 | No |  | No | Yes | No |
| 27 | 2013 | 84 | Female | Blood | 1b | 87 | 87 | No |  | No | No | No |
| 28 | 2013 | 75 | Female | Blood | 1b | 3 | 3 | No |  | Yes | No | No |
| 29 | 2013 | 60 | Male | CSF | 4b | 1 | 1 | No |  | No | No | Yes |
| 30 | 2013 | 72 | Male | Blood | 4b | 4 | 4 | No |  | Yes | No | No |
| 31 | 2013 | 31 | Female | Blood | 1b | 87 | 87 | No |  | No | Yes | No |
| 32 | 2013 | 54 | Female | Blood | 1a | 431 | 101 | No |  | No | No | No |
| 33 | 2013 | 34 | Female | Placenta | 1b | 87 | 87 | No |  | No | Yes | No |
| 34 | 2013 | 0 | Male | Blood | 1b | 87 | 87 | No |  | No | Yes | No |
| 35 | 2013 | 85 | Male | Blood | 4b | 1 | 1 | Yes | One year | Yes | No | No |
| 36 | 2013 | 78 | Male | Blood | 1b | 87 | 87 | Yes | One month | No | No | No |
| 37 | 2013 | 54 | Male | Blood | 4b | 4 | 4 | No |  | Yes | No | No |
| 38 | 2013 | 45 | Female | Blood | 4b | 6 | 6 | No |  | Yes | No | No |
| 39 | 2013 | 30 | Female | Blood | 4b | 1 | 1 | No |  | No | Yes | No |
| 40 | 2013 | 78 | Female | Blood | 1b | 87 | 87 | No |  | No | No | No |
| 41 | 2014 | 33 | Female | Blood | 1b | 87 | 87 | No |  | No | Yes | No |
| 42 | 2014 | 81 | Male | Blood | 1b | 87 | 87 | Yes | One year | Yes | No | No |
| 43 | 2014 | 34 | Female | Faeces | 1b | 87 | 87 | No |  | Yes | No | No |
| 44 | 2014 | 91 | Male | Blood | 1a | 8 | 8 | Yes | One month | No | No | No |
| 45 | 2014 | 28 | Female | Placenta | 1b | 87 | 87 | No |  | No | Yes | No |
| 46 | 2014 | 0 | Male | Otic | 1b | 87 | 87 | No |  | No | Yes | No |
| 47 | 2014 | 69 | Male | Aqueous humour | 4b | 1 | 1 | No |  | No | No | No |
| 48 | 2014 | 74 | Male | CSF | 4b | 219 | 4 | No |  | No | No | Yes |
| 49 | 2014 | 73 | Male | Blood | 1a | 37 | 37 | Yes | One month | Yes | No | No |
| 50 | 2014 | 58 | Male | CSF | 4b | 219 | 4 | Yes | One year | Yes | No | Yes |
| 51 | 2015 | 90 | Male | Blood | 4b | 1 | 1 | No |  | No | No | No |
| 52 | 2015 | 54 | Female | Blood | 4b | 1 | 1 | No |  | No | No | No |
| 53 | 2015 | 29 | Female | Blood | 4b | 1 | 1 | No |  | No | Yes | No |
| 54 | 2015 | 65 | Male | Blood | 4b | 1 | 1 | No |  | No | No | No |
| 55 | 2015 | 59 | Male | Urine | 4b | 1 | 1 | No |  | No | No | No |
| 56 | 2015 | 64 | Female | Blood | 4b | 213 | 213 | Yes | One month | Yes | No | No |
| 57 | 2015 | 66 | Male | Peritoneal fluid | 4b | 4 | 4 | No |  | No | No | No |
| 58 | 2016 | 88 | Female | Blood | 1b | 3 | 3 | Yes | One year | Yes | No | No |
| 59 | 2016 | 89 | Female | Intervertebral disc | 4b | 213 | 213 | No |  | No | No | No |
| 60 | 2016 | 74 | Male | CSF | 4b | 219 | 4 | No |  | No | No | Yes |
| 61 | 2016 | 77 | Male | Blood | 1b | 3 | 3 | Yes | One month | Yes | No | No |
| 62 | 2016 | 73 | Male | Blood | 4b | 219 | 4 | Yes | One month | Yes | No | Yes |
| 63 | 2016 | 81 | Male | Blood | 1b | 3 | 3 | No |  | Yes | No | No |
| 64 | 2016 | 80 | Male | Blood | 1a | 431 | 101 | No |  | Yes | No | No |
| 65 | 2016 | 82 | Male | Blood | 1a | 431 | 101 | No |  | Yes | No | No |
| 66 | 2016 | 80 | Male | CSF | 1a | 431 | 101 | No |  | No | No | Yes |
| 67 | 2017 | 33 | Female | CSF | 1b | 82 | 82 | No |  | Yes | No | Yes |
| 68 | 2017 | 77 | Female | Blood | 1b | 3 | 3 | Yes | One month | No | No | No |
| 69 | 2017 | 47 | Male | Blood | 1a | 155 | 155 | No |  | Yes | No | No |
| 70 | 2017 | 54 | Female | CSF | 1a | 8 | 8 | Yes | One month | Yes | No | Yes |
| 71 | 2017 | 33 | Female | Placenta | 4b | 1 | 1 | No |  | No | Yes | No |
| 72 | 2018 | 31 | Female | Placenta | 4b | 1 | 1 | No |  | No | Yes | No |
| 73 | 2018 | 77 | Male | Blood | 1a | 155 | 155 | No |  | Yes | No | Yes |
| 74 | 2018 | 78 | Male | Blood | 4b | 388 | 388 | Yes | One month | Yes | No | No |
| 75 | 2018 | 81 | Male | Blood | 4b | 1 | 1 | No |  | No | No | Yes |
| 76 | 2018 | 74 | Male | Blood | 1a | 8 | 8 | Yes | One month | Yes | No | No |
| 77 | 2018 | 78 | Female | Blood | 4b | 219 | 4 | Yes | One year | Yes | No | Yes |
| 78 | 2018 | 81 | Male | Blood | 4b | 1 | 1 | Yes | One year | Yes | No | No |
| 79 | 2018 | 80 | Male | Blood | 4b | 1 | 1 | No |  | No | No | No |
| 80 | 2018 | 79 | Male | CSF | 4b | 6 | 6 | Yes | One year | No | No | Yes |
| 81 | 2018 | 84 | Male | Blood | 1a | 37 | 37 | No |  | No | No | No |
| 82 | 2018 | 91 | Male | Blood | 4b | 1 | 1 | No |  | No | No | No |
| 83 | 2019 | 40 | Female | Placenta | 4b | 1 | 1 | No |  | No | Yes | No |
| 84 | 2019 | 38 | Female | Placenta | 4b | 1 | 1 | No |  | No | Yes | No |
| 85 | 2019 | 51 | Male | Blood | 1a | 8 | 8 | No |  | Yes | No | No |
| 86 | 2019 | 87 | Female | Blood | 1b | 3 | 3 | No |  | No | No | No |
| 87 | 2019 | 80 | Male | Blood | 4b | 6 | 6 | No |  | No | No | No |
| 88 | 2020 | 67 | Male | Blood | 4b | 2 | 2 | Yes | One year | Yes | No | No |
| 89 | 2020 | 78 | Male | CSF | 4b | 219 | 4 | Yes | One year | Yes | No | Yes |
| 90 | 2020 | 44 | Male | CSF | 4b | 1 | 1 | No |  | No | No | Yes |
| 91 | 2020 | 79 | Female | Blood | 4b | 388 | 388 | No |  | Yes | No | No |
| 92 | 2020 | 32 | Female | Blood | 4b | 1 | 1 | No |  | No | Yes | No |
| 93 | 2020 | 83 | Female | Blood | 1a | 29 | 29 | Yes | One month | No | No | No |

a: Sequence Type; b: Clonal Complex; c: death in less than a year since diagnosis of listeriosis; d: less than one month or less than one year; empty cells represent more than one year survival.
