# Supplementary material for: Epidemiology and Clinical Features of Listeriosis in Gipuzkoa, Spain, 2010–2020
Source: Front Microbiol. 2022 Jun 9;13:894334. doi: 10.3389/fmicb.2022.894334 (PMC9218358; doi:10.3389/fmicb.2022.894334)
Supplement: Supplementary file 2 [file Table_2.docx]

Table S2. Correspondence between STs and serotypes

| Serotype | ST1 | ST2 | ST3 | ST4 | ST6 | ST8 | ST9 | ST14 | ST16 | ST18 | ST29 | ST37 | ST54 | ST82 | ST87 | ST155 | ST194 | ST213 | ST217 | ST219 | ST379 | ST388 | ST431 | ST2892 |
| --- | --- | --- | --- | --- | --- | --- | --- | --- | --- | --- | --- | --- | --- | --- | --- | --- | --- | --- | --- | --- | --- | --- | --- | --- |
| Serotype 4b | 27 | 2 | 0 | 4 | 7 | 0 | 0 | 0 | 0 | 0 | 0 | 0 | 1 | 0 | 0 | 0 | 1 | 4 | 1 | 9 | 0 | 2 | 0 | 1 |
| Serotype 1/2b | 0 | 0 | 8 | 0 | 0 | 0 | 0 | 0 | 0 | 0 | 0 | 0 | 0 | 1 | 21 | 0 | 0 | 0 | 0 | 0 | 0 | 0 | 0 | 0 |
| Serotype 1/2a | 0 | 0 | 0 | 0 | 0 | 7 | 0 | 1 | 1 | 1 | 1 | 3 | 0 | 0 | 0 | 2 | 0 | 0 | 0 | 0 | 1 | 0 | 4 | 0 |
| Serotype 1/2c | 0 | 0 | 0 | 0 | 0 | 0 | 1 | 0 | 0 | 0 | 0 | 0 | 0 | 0 | 0 | 0 | 0 | 0 | 0 | 0 | 0 | 0 | 0 | 0 |
