# Supplementary material for: Epidemiology and Clinical Features of Listeriosis in Gipuzkoa, Spain, 2010–2020
Source: Front Microbiol. 2022 Jun 9;13:894334. doi: 10.3389/fmicb.2022.894334 (PMC9218358; doi:10.3389/fmicb.2022.894334)
Supplement: Supplementary file 3 [file Table_3.docx]

Table S3. Total cases and STs per year

|  | Year | Cases per year | ST1 | ST2 | ST3 | ST4 | ST6 | ST8 | ST9 | ST14 | ST16 | ST18 | ST29 | ST37 | ST54 | ST82 | ST87 | ST155 | ST194 | ST213 | ST217 | ST219 | ST379 | ST388 | ST431 | ST2892 |
| --- | --- | --- | --- | --- | --- | --- | --- | --- | --- | --- | --- | --- | --- | --- | --- | --- | --- | --- | --- | --- | --- | --- | --- | --- | --- | --- |
|  | 2010 | 7 | 0 | 1 | 1 | 0 | 1 | 1 | 0 | 0 | 0 | 0 | 0 | 0 | 0 | 0 | 3 | 0 | 0 | 0 | 0 | 0 | 0 | 0 | 0 | 0 |
|  | 2011 | 10 | 2 | 0 | 0 | 1 | 0 | 0 | 0 | 1 | 0 | 1 | 0 | 0 | 0 | 0 | 2 | 0 | 0 | 2 | 1 | 0 | 0 | 0 | 0 | 0 |
|  | 2012 | 9 | 3 | 0 | 0 | 0 | 1 | 0 | 1 | 0 | 0 | 0 | 0 | 0 | 1 | 0 | 1 | 0 | 0 | 0 | 0 | 2 | 0 | 0 | 0 | 0 |
|  | 2013 | 18 | 3 | 0 | 1 | 2 | 1 | 1 | 0 | 0 | 1 | 0 | 0 | 0 | 0 | 0 | 7 | 0 | 1 | 0 | 0 | 0 | 0 | 0 | 1 | 0 |
|  | 2014 | 14 | 1 | 0 | 0 | 0 | 1 | 1 | 0 | 0 | 0 | 0 | 0 | 1 | 0 | 0 | 7 | 0 | 0 | 0 | 0 | 2 | 0 | 0 | 0 | 1 |
|  | 2015 | 9 | 5 | 0 | 1 | 1 | 0 | 0 | 0 | 0 | 0 | 0 | 0 | 1 | 0 | 0 | 0 | 0 | 0 | 1 | 0 | 0 | 0 | 0 | 0 | 0 |
|  | 2016 | 10 | 0 | 0 | 3 | 0 | 0 | 0 | 0 | 0 | 0 | 0 | 0 | 0 | 0 | 0 | 1 | 0 | 0 | 1 | 0 | 2 | 0 | 0 | 3 | 0 |
|  | 2017 | 6 | 2 | 0 | 1 | 0 | 0 | 1 | 0 | 0 | 0 | 0 | 0 | 0 | 0 | 1 | 0 | 1 | 0 | 0 | 0 | 0 | 0 | 0 | 0 | 0 |
|  | 2018 | 14 | 6 | 0 | 0 | 0 | 2 | 1 | 0 | 0 | 0 | 0 | 0 | 1 | 0 | 0 | 0 | 1 | 0 | 0 | 0 | 1 | 1 | 1 | 0 | 0 |
|  | 2019 | 8 | 3 | 0 | 1 | 0 | 1 | 2 | 0 | 0 | 0 | 0 | 0 | 0 | 0 | 0 | 0 | 0 | 0 | 0 | 0 | 1 | 0 | 0 | 0 | 0 |
|  | 2020 | 6 | 2 | 1 | 0 | 0 | 0 | 0 | 0 | 0 | 0 | 0 | 1 | 0 | 0 | 0 | 0 | 0 | 0 | 0 | 0 | 1 | 0 | 1 | 0 | 0 |
|  | Total | 111 | 27 | 2 | 8 | 4 | 7 | 7 | 1 | 1 | 1 | 1 | 1 | 3 | 1 | 1 | 21 | 2 | 1 | 4 | 1 | 9 | 1 | 2 | 4 | 1 |
